# Characterization of the hypersensitive response‐like cell death phenomenon induced by targeting antiviral lectin griffithsin to the secretory pathway

**DOI:** 10.1111/pbi.12917

**Published:** 2018-05-02

**Authors:** Bo Min Kim, Hester Catharina Therese Lotter‐Stark, Edward P. Rybicki, Rachel K. Chikwamba, Kenneth E. Palmer

**Affiliations:** ^1^ Center for Predictive Medicine James Graham Brown Cancer Center Department of Pharmacology and Toxicology University of Louisville School of Medicine Louisville KY USA; ^2^ Biosciences Council for Scientific and Industrial Research (CSIR) Pretoria South Africa; ^3^ Department of Molecular & Cell Biology Institute of Infectious Disease and Molecular Medicine University of Cape Town Cape Town South Africa

**Keywords:** griffithsin (GRFT), human immunodeficiency virus (HIV), lectin, hypersensitive response (HR)

## Abstract

Griffithsin (GRFT) is an antiviral lectin, originally derived from a red alga, which is currently being investigated as a topical microbicide to prevent transmission of human immunodeficiency virus (HIV). Targeting GRFT to the apoplast for production in *Nicotiana benthamiana* resulted in necrotic symptoms associated with a hypersensitive response (HR)‐like cell death, accompanied by H_2_O_2_ generation and increased PR1 expression. Mannose‐binding lectins surfactant protein D (SP‐D), cyanovirin‐N (CV‐N) and human mannose‐binding lectin (hMBL) also induce salicylic acid (SA)‐dependent HR‐like cell death in *N. benthamiana*, and this effect is mediated by the lectin's glycan binding activity. We found that secreted GRFT interacts with an endogenous glycoprotein, α‐xylosidase (XYL1), which is involved in cell wall organization. The necrotic effect could be mitigated by overexpression of Arabidopsis XYL1, and by co‐expression of SA‐degrading enzyme NahG, providing strategies for enhancing expression of oligomannose‐binding lectins in plants.

## Introduction

Griffithsin (GRFT) is a lectin originally isolated from a red algae, *Griffithsia* sp (Mori *et al*., 2005). GRFT is one of the most potent HIV‐1 entry inhibitors yet described, and hence of great interest for use in HIV‐1 prophylaxis and therapy (Lusvarghi and Bewley, 2016; O'Keefe *et al*., 2009). We developed an efficient and scalable manufacturing method for recombinant GRFT using a tobamovirus‐based gene expression system in *Nicotiana benthamiana* and have shown that the plant produced GRFT product has a favourable preclinical safety and efficacy profile, supporting its development as a topical microbicide to prevent HIV‐1 and HSV‐2 transmission (Kouokam *et al*., 2011, 2016; Nixon *et al*., 2013; O'Keefe *et al*., 2009). Beyond its use for prevention of viral sexually transmitted infection, recent studies showed GRFT has antiviral activity against enveloped viruses such as hepatitis C virus (HCV), SARS coronavirus and Japanese encephalitis virus (Ishag *et al*., 2013; Meuleman *et al*., 2011; O'Keefe *et al*., 2010). Prolonging the serum half‐life of biologics is generally desirable for therapeutic use, and protein engineering strategies such as creation of immunoadhesins, serum albumin fusions and pegylation have been used to improve pharmacokinetics (Strohl, 2015). Although we express wild‐type GRFT in the cytoplasm of *N. benthamiana* plants, achieving impressive yields, protein engineering strategies for improving serum half‐life will often require directing fusion proteins to the secretory pathway to achieve proper folding and post‐translational modifications. We were aware that targeting GRFT to the secretory pathway in *N. benthamiana* causes severe necrotic symptoms and consequently low yields of protein (Stark, 2013), and therefore decided to investigate the molecular basis of the host response to ectopic expression of lectins in the secretory pathway.

Griffithsin is a ~13 kDa domain‐swapped homodimer of which the first 18 amino acids of one monomer join the other monomer in forming a β‐prism of three four‐stranded sheets (O'Keefe *et al*., 2009). The GRFT homodimer has six carbohydrate‐binding sites, three located at each of the opposite ends of the double‐prism homodimer (Moulaei *et al*., 2010). GRFT binds to high‐mannose N‐glycans on the HIV viral envelope spike gp120 and inhibits the viral envelope structural transitions which are essential for viral entry, thus preventing HIV infection of T cells (Mori *et al*., 2005). GRFT also inhibits cell‐associated HIV‐1 infection of dendritic cells (DC) by inhibiting the transfer of the virus from DC‐SIGN to uninfected T cells. Replacement of an aspartic acid residue in each of the glycan binding sites abrogates GRFT's ability to bind oligomannose glycosylated proteins and eliminates its viral neutralization activity (Xue *et al*., 2012). Therefore, the GRFT activity is dependent on its ability to bind multiple mannose N‐glycans.

In preliminary studies, we found that targeting GRFT to the secretory pathway induced a strong necrotic phenotype reminiscent of the hypersensitive reaction (HR), a resistance response induced by interaction between a plant resistance gene (R gene) and an avirulence factor of a pathogen (Avr gene). HR is a type of programmed cell death that accompanies defence reactions such as generation of reactive oxygen species (ROS) including H_2_O_2_, the accumulation of plant hormones such as salicylic acid (SA), jasmonic acid (JA) or ethylene, and the expression of pathogenesis‐related (PR) genes (Hammond‐Kosack and Jones, 1997; Nurnberger *et al*., 2004). The HR signals extend to noninfected cells and lead to systemic acquired resistance that is effective against a broad‐spectrum of pathogens (Durrant and Dong, 2004). In most HR, SA signalling is involved and SA accumulation shows a strong correlation with generation of ROS (Durrant and Dong, 2004; Lee and Park, 2010). Naphthalene hydroxylase (NahG), which is derived from *Pseudomonas syringae*, degrades SA and suppresses SA‐regulated signalling (Brodersen *et al*., 2005; Clarke *et al*., 2004). NahG overexpression inhibits SA‐dependent HR induction in various plant species (Brodersen *et al*., 2005; Mur *et al*., 1997).

In this study, we expressed GRFT fused with subcellular localization signals using the magnICON replicon‐based expression system (Giritch *et al*., 2006) to avoid any potentially confounding pathogenesis signalling that involve the tobamovirus coat protein, but also to achieve expression levels comparable to the recombinant TMV vector system we used previously (O'Keefe *et al*., 2009, 2010). Our preliminary data showed that GRFT fused with an apoplast localization signal caused severe laminar necrosis, despite low accumulation levels of apoplast‐targeted GRFT compared with cytosol‐localized GRFT. Here, we analysed the mechanism whereby GRFT induces HR‐like cell death in *N. benthamiana*. We show GRFT‐induced HR‐like cell death is SA‐dependent. Analysis of expression of the GRFT mutant showed six carbohydrate‐binding sites per GRFT dimer were required for the induction of HR‐like cell death and provided data supporting our hypothesis that a specific interaction between GRFT and an apoplast‐located endogenous glycoprotein, XYL1, initiates the HR response. Molecular characterization of the protein interactions and physiological response to targeting GRFT expression to the apoplast helped us identify strategies to dampen the necrosis response and rescue expression of GRFT‐ and similar engineered lectin fusions in the plant secretory pathway.

## Results

### Effect of SA accumulation on GRFT protein expression

Griffithsin localized in the cytosol (GRFT‐Cyt) expressed by magnICON showed a higher yield relative to GRFT fused with an apoplast subcellular localization signal (Figure S1a). Accumulation levels of GRFT localized in apoplast (GRFT‐Apo) were markedly lower. Targeting GRFT expression to the apoplast caused necrosis in *N. benthamiana*, which was not observed in the plants expressing GRFT in cytosol (Figure S1d). As another group had shown that overexpression of plant lectin CaMBL1 caused salicylic acid (SA)‐dependent HR‐like cell death in *N. benthamiana* (Hwang and Hwang, 2011), we hypothesized that a similar phenomenon could account for the GRFT‐induced necrosis. Indeed, the GRFT‐Apo‐induced necrosis was found to be associated with an increase in H_2_O_2_ production as indicated by staining by 2′7′‐dichlorofluorescein diacetate staining (H2DCFDA, Figure S1b). Furthermore, Evans blue staining showed that cell death occurred in the regions with induced H_2_O_2_ production (Figure S1b, Kim *et al*., 2008). As H_2_O_2_ acts an inducer of defence‐associated genes such as pathogenesis‐related protein (PR) genes, SA‐dependent PR1 gene expression levels were analysed. Quantitative real‐time PCR showed that PR1 gene expression was increased (Figure S1c). No necrotic symptoms, elevated H_2_O_2_ levels or enhanced PR1 gene expression were observed in the control plants (empty vector) or in plants expressing GRFT‐Cyt. Similarly, expression of GRFT‐Apo by a plant expression binary vector, pTRA (nonreplicating), caused necrosis symptoms, resulting in a lower accumulation level than GRFT‐Cyt. These results confirm that the necrotic phenotype is due to apoplast targeting of GRFT and that the tobacco mosaic virus proteins used to amplify RNA levels in the magnICON system are not involved in GRFT‐Apo‐induced necrosis (Figure S3).

**Figure 1:**
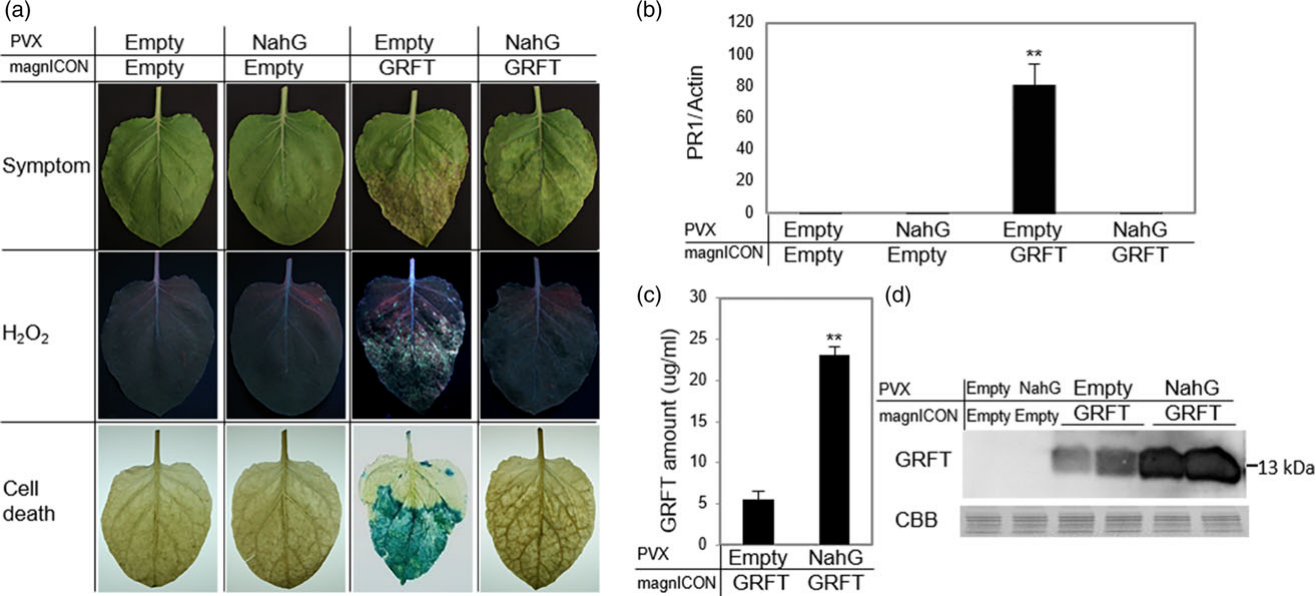
NahG expression suppressed GRFT‐induced cell death in *Nicotiana benthamiana*. (a) Necrosis induction, H_2_O_2_ generation and enhancement of PR1 gene expression by GRFT were inhibited by NahG expression in *N. benthamiana*. Detection of cell death and H_2_O_2_ generation in the inoculated plant leaves. Dead cells were stained by Evans blue, and H_2_O_2_ was detected using the H_2_O_2_‐sensitive fluorescent probe H2DCFDA. (b) Analysis of PR1 expression levels by real‐time RT‐PCR. The PR1 mRNA levels relative to the actin mRNA levels were shown. PVX‐empty + mganICON‐empty, PVX‐NahG + magnICON‐empty, PVX‐empty + magnICON‐GRFT and PVX‐NahG + magnICON‐GRFT were vacuum agro‐infiltrated into *N. benthamiana*. Leaves were harvested 5 days after infiltration and analysed. **P <* *0.01 asterisks indicate significant difference [one‐way ANOVA with Bonferroni's multiple comparison test (*n *=* *3)] between inoculated plants group. Error bars represent standard errors of the means. (c) Measurement of GRFT accumulation levels by ELISA. HIV gp‐120 was bound to the wells of a 96‐well plate and subsequently incubated with total protein from the plants agro‐infiltrated with PVX‐empty + magnICON‐GRFT or PVX‐NahG + magnICON‐GRFT. After visualized by HRP‐labelled anti‐GRFT antibody, OD was measured by absorbance at 450 nm. **P <* *0.01, asterisks indicate significant difference [Student's *t*‐test (*n *=* *3)] between PVX‐empty + magnICON‐GRFT and PVX‐NahG + magnICON‐GRFT inoculated plants. Error bars represent standard errors of the means. (d) Measurement of GRFT accumulation levels by Western blotting. Anti‐GRFT (rabbit) and anti‐rabbit HRP were used for detection. CBB stain indicates loaded protein amounts are equal.

PR1 is a marker for salicylic acid‐mediated plant defence. PR1′s induction indicated the possibility that SA accumulation was involved in GRFT‐Apo's induction of HR‐like cell death. To test this hypothesis, we used NahG, a protein from *Pseudomonas syringae* which degrades SA (Brodersen *et al*., 2005). We cloned NahG into a PVX replicon vector (Figure S2, Giritch *et al*., 2006). Concomitant analyses of PR1 expression and H_2_O_2_ levels demonstrated that control plants (transiently expressing GRFT alone) supported high levels of PR1 gene expression and H_2_O_2_ generation (Figure 1a,b). Conversely, plants that were inoculated with PVX‐NahG and magnICON‐GRFT exhibited low levels of PR1 gene expression and we could not detect H_2_O_2_ generation in such plants (Figure 1a,b). Therefore, GRFT‐Apo‐induced HR‐like cell death required SA accumulation, and the inhibition of SA accumulation by NahG suppressed the symptoms.

Our results to this point suggest that expression of NahG suppressed HR‐related cell death and consequently may allow the co‐expressed GRFT protein to accumulate to higher levels in the healthier tissue. To validate this hypothesis, functional GRFT protein levels were measured using an HIV‐1 gp120‐binding enzyme‐linked immunosorbent (ELISA) and total GRFT amounts were assessed by Western blotting (O'Keefe *et al*., 2009). The ELISA and Western blot analysis showed that both functional and gross GRFT expression levels are substantially increased by NahG expression (Figure 1c,d). This result suggests that overexpressing NahG in plants may enhance GRFT production and that a NahG transgenic host may facilitate transient expression of secreted GRFT products.

### GRFT carbohydrate‐binding domains induces HR

**Figure 2:**
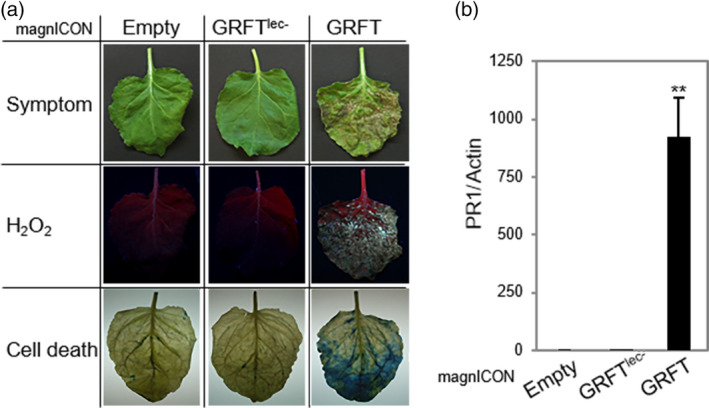
The loss of carbohydrate‐binding sites in GRFT inhibits GRFT‐induced cell death in *N. benthamiana*. magnICON‐mutated GRFT (GRFT ^lec‐^), which contains the substitutions D30A, D70A and D112A in carbohydrate‐binding sites, was agro‐infiltrated into *N. benthamiana*. (a) Detection of cell death and H_2_O_2_ was performed using H2DCFDA and Evans blue in magnICON‐GRFT ^lec‐^ infiltrated *N. benthamiana* 5 days after infiltration. Cell death and H_2_O_2_ were not detected in magnICON‐GRFT ^lec‐^ infiltrated plants. (b) Analysis of PR1 expression levels by real‐time RT‐PCR. The PR1 mRNA levels relative to the actin mRNA levels were shown. Empty magnICON vector, magnICON‐GRFT or mganICON‐GRFT ^lec‐^, was vacuum agro‐infiltrated into *N. benthamiana*. Leaves were harvested 5 days after infiltration and analysed. **P <* *0.01, asterisks indicate significant difference [one‐way ANOVA with Bonferroni's multiple comparison test (*n *=* *3)] between inoculated plants group. Error bars represent standard errors of the means.

GRFT's activity is due to its specific binding of high‐mannose N‐glycans. Mutations at the carbohydrate‐binding site in GRFT disrupt its ability to bind gp120 and inhibit HIV infection (Moulaei *et al*., 2010). Based on this feature, we hypothesized that GRFT's high mannose binding activity is involved in induction of necrosis. To determine whether the carbohydrate‐binding sites in GRFT are required for the induction of HR‐like cell death, we created a mutated GRFT (GRFT^lec‐^), which contains the amino acid substitutions D30N, D70N and D112N in the carbohydrate‐binding sites, abolishing lectin activity. GRFT and GRFT^lec‐^ fused to the apoplast localization signal were expressed by agroinfiltration. Expression of wild‐type GRFT‐Apo caused severe cell death accompanied by H_2_O_2_ generation, which was not detected in GRFT^lec‐^‐Apo‐expressing plants (Figure 2a). PR1 expression also decreased in GRFT^lec‐^‐expressing plants (Figure 2b). Co‐inoculation with NahG and GRFT^lec‐^‐Apo did not show any changes in necrosis as compared to PVX‐empty and magnICON‐GRFT^lec‐^ (Figure S4a,b). These results indicate that the aspartic acid residues responsible for GRFT's high mannose binding also play an important role in causing cell death when the protein is expressed in the apoplast of *N. benthamiana*.

### Purified GRFT can induce HR

To determine whether the presence of GRFT protein is sufficient to induce HR‐like cell death, and to rule out any confounding effect of viral replication on the GRFT‐mediated necrotic phenotype, we infiltrated purified GRFT or GRFT^lec‐^ protein into 2‐week‐old *N. benthamiana* plants using vacuum infiltration. 5 days after GRFT protein infiltration, the *N. benthamiana* plants showed necrotic symptoms (Figure S5a). GRFT^lec‐^ protein or buffer‐infiltrated plants did not show any symptoms. We detected H_2_O_2_ signal and cell death only in the GRFT‐infiltrated plants. PR1 gene expression level was also increased in the GRFT‐infiltrated plants (Figure S5b). These results show that GRFT protein with intact lectin activity alone is sufficient to induce HR‐like cell death even in the absence of any interaction with a viral factor in the magnICON vector.

### Other mannose binding lectins also induce HR when targeted to the apoplast

To test whether other mannose‐binding lectins induce HR‐like cell death in *N. benthamiana* as observed in GRFT‐Apo expression, we focused on surfactant protein D (SP‐D, Hasegawa *et al*., 2015), cyanovirin‐N (CV‐N, Gao *et al*., 2010; Xiong *et al*., 2010), human mannose‐binding lectin (hMBL, Ibernon *et al*., 2014) and galectin‐9 (Gal‐9, Arikawa *et al*., 2010; Wang *et al*., 2008) to express in *N. benthamiana* plants. SP‐D, CV‐N and hMBL are classified as high‐mannose‐binding lectins, whereas Gal‐9 is a galactose‐binding lectin. CV‐N is isolated from the cyanobacterium *Nostoc elipsopsorum* (Boyd *et al*., 1997), and the other lectins are derived from humans. We set out to understand why mannose‐binding lectins induce necrosis and determine whether there is a common mechanism involved. This would be a logical first step to engineer a more efficient production system for GRFT and other pharmaceutical lectin proteins in plant.

**Figure 3:**
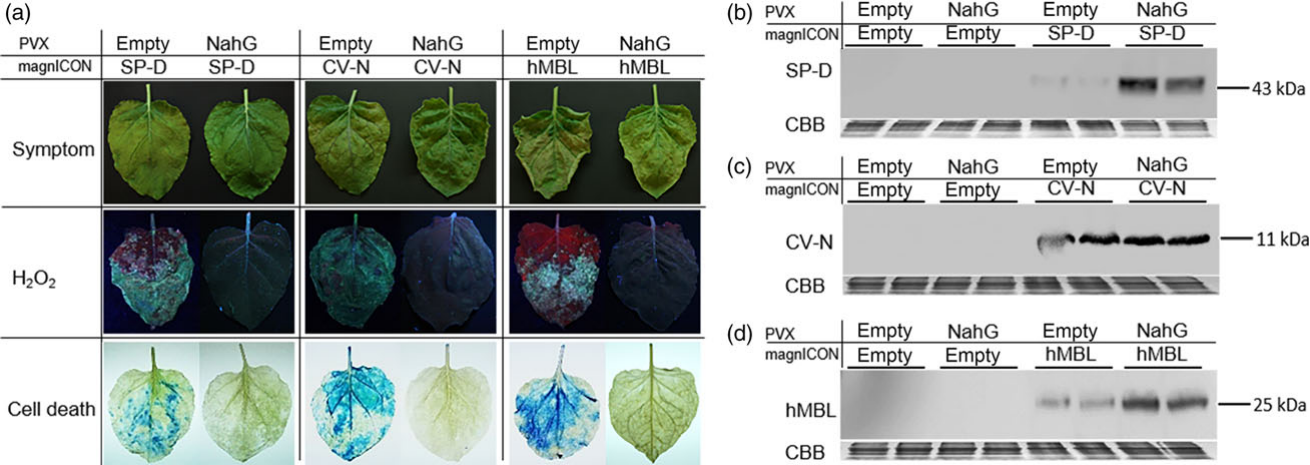
NahG expression suppressed carbohydrate‐binding lectins‐induced cell death in *N. benthamiana*. (a) Necrosis induction, H_2_O_2_ generation and cell death by the carbohydrate binding lectins were inhibited by NahG expression in *N. benthamiana*. Detection of cell death and H_2_O_2_ generation in the inoculated plant leaves. Dead cells were stained by Evans blue, and H_2_O_2_ was detected using the H_2_O_2_‐sensitive fluorescent probe H2DCFDA. (b–d) Measurement of the lectins’ accumulation levels by Western blotting. Anti‐His (rabbit) and anti‐rabbit HRP were used for detection. CBB stain indicates loaded protein amounts are equal.

Plant codon‐optimized SP‐D, CV‐N, hMBL and Gal‐9 sequences were cloned into the magnICON vector with the apoplast signal peptide. The lectins were expressed in *N. benthamiana* by agroinfiltration. When the lectins were fused with an apoplast‐targeting signal, all three mannose‐binding lectins induced necrotic symptoms, but plants expressing Gal‐9 did not (Figure S6a). In the necrotic plants, cell death, H_2_O_2_ generation and increased PR1 expression levels were detected as in GRFT‐Apo‐induced necrosis (Figure S6a,b). To investigate whether the necrosis by the mannose‐binding lectins is dependent on the SA pathway as shown with GRFT, we suppressed the SA signal pathway using NahG. NahG and the mannose‐binding lectins were co‐inoculated into *N. benthamiana*. The co‐expression of NahG and each mannose binding lectin inhibited cell death and H_2_O_2_ generation (Figure 3a). We also detected the amount of the lectin expression in the plants by Western blot. PVX‐NahG expressed with magnICON‐SP‐D and magnICON‐hMBL, respectively, inoculated plants expressed more protein than magnICON‐SP‐D and magnICON‐hMBL (Figure 3b). However, the PVX‐NahG and magnICON‐CV‐N inoculated plants expressed the same level of protein. Like GRFT, these secreted mannose‐binding lectins induce SA‐dependent HR‐like cell death, which could be rescued by NahG expression. Moreover, NahG could boost protein expression in some cases.

**Figure 4:**
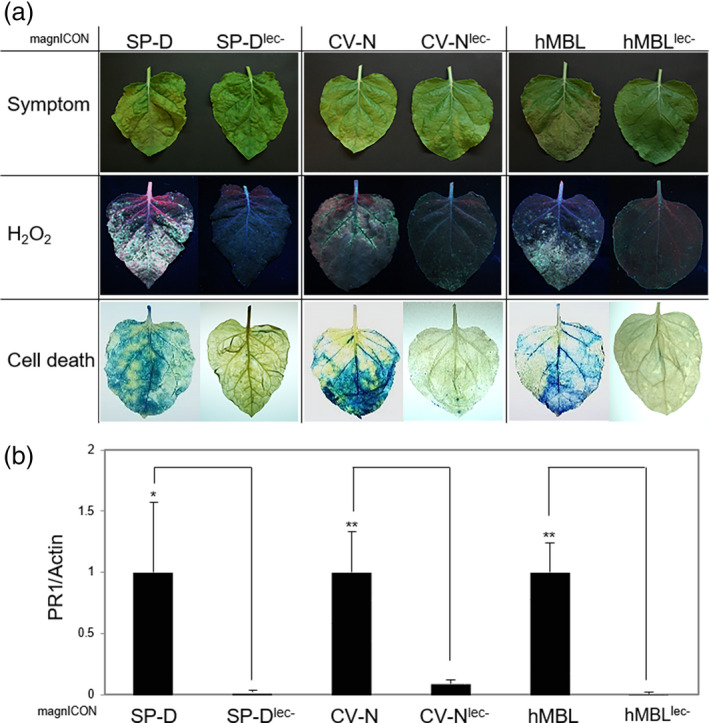
The loss of carbohydrate‐binding sites in SP‐D, CV‐N, hMBL inhibits cell death in *N. benthamiana*. (a) Detection of cell death and H_2_O_2_ was performed using H2DCFDA and Evans blue in magnICON‐SP‐D^lec‐^(E321Q and N323D), magnICON‐CV‐N^lec‐^(K3N, Y7A, E23I and N93A), magnICON‐hMBL ^lec‐^(G51E, R52C, G54D, G57E and G60E) infiltrated *N. benthamiana* 5 days after infiltration. Cell death and H_2_O_2_ were not detected in mutated lectin (SP‐D^lec‐^, CV‐N^lec‐^ or hMBL ^lec‐^) infiltrated plants. (b) Analysis of PR1 expression levels by real‐time RT‐PCR. The PR1 mRNA levels relative to the actin mRNA levels were shown. Wild‐type lectins of lectin deficient of SP‐D, CV‐N and hMBL were vacuum agro‐infiltrated with *N. benthamiana*. Leaves were harvested 5 days after the infiltration to analysis PR1 gene accumulation in early stage. **P <* *0.01 and *P <* *0.05, asterisks indicate significant difference [Student's *t*‐test (*n *=* *3)] between wild‐type lectin and lectin deficient ‐ inoculated plants group. All test samples were measured in triplicate.

As we showed, GRFT's high‐mannose binding ability has an important role in the induction of cell death. We investigated whether SP‐D, CV‐N and hMBL use the same mechanism as GRFT to induce HR‐like cell death through their mannose‐binding activity. SP‐D, CV‐N and hMBL are well‐studied lectins, and the amino acid substitutions which disrupt their mannose binding activities eliminate their pharmacological effects (Larsen *et al*., 2004; Liu *et al*., 2009; Ogasawara and Voelker, 1995). To test whether the mannose binding activities in SP‐D, CV‐N and hMBL also induce HR‐like cell death in *N. benthamiana,* we engineered lectin activity‐deficient SP‐D, CV‐N and hMBL by substituting alanine for the specific amino acid residues involved in glycan binding (see Experimental procedures). The mutated lectins, fused with the apoplast signal, were expressed in *N. benthamiana* using the agrobacterium‐mediated magnICON system. The mutated lectin accumulation levels in *N. benthamiana* were confirmed by Western blot (Figure S7). The accumulation levels of lectins and their mutated allele differed in the Western blot analysis. The mutated lectins did not induce necrotic symptoms in *N. benthamiana* plants, and PR1 expression activation and H_2_O_2_ generation were not detected (Figure 4a,b). These results indicate that, as for GRFT, the high‐mannose N‐glycan binding activity in SP‐D, CV‐N and hMBL is essential for cell death induction in plants.

### GRFT–plant protein interactions detected by in situ PLA system

To investigate the possibility that GRFT‐Apo interacts with a plant protein localized in the apoplast and that this specific interaction leads to HR‐like cell death, interaction of GRFT with one or more plant proteins was analysed by in situ proximity ligation assay (PLA), a method to detect proteins using two primary antibodies and quantification of specific protein‐protein interactions in situ (Soderberg *et al*., 2008). We created mouse antibodies against *N. benthamiana* total proteins and secreted proteins. These antibodies were confirmed to recognize *N. benthamiana* proteins by immunostaining (Figure S11). First, we evaluated the GRFT–plant protein interactions using mouse antibody against total proteins and goat antibody against GRFT. Plants expressing GRFT‐Apo showed a strong PLA signal, and this signal localized in the plant membrane (Figure S8). On the other hand, there was no signal detected in either the empty vector or GRFT^lec‐^ infiltrated samples.

Next, we evaluated interactions between secreted GRFT and secreted plant proteins. Again, only the plants expressing GRFT‐Apo, and not GRFT^lec‐^‐Apo or empty vector, displayed signal indicating ligation of proximal proteins. This signal localized to the space outside the plant cells. These results indicate that GRFT is interacting with plant proteins via its carbohydrate‐binding domain. We speculate this secreted GRFT–host protein interaction plays an important role in its HR‐like cell death induction.

To analyse the mechanism by which secreted GRFT induces HR‐like cell death, we attempted to isolate host factors that interact with GRFT using an Arabidopsis cDNA library (Clontech) in a yeast two‐hybrid assay. We screened independent yeast transformants and obtained partial XYL1 (*AtXYL1*, AT1G68560) fragments as a candidate GRFT‐interacting host factor. AtXYL1 (At1g68560) was identified an α‐xylosidase against xyloglucan (XG), which localized in cell wall and apoplast (Sampedro *et al*., 2010). AtXYL1 is a 915‐amino acid protein with eight N‐glycosylation sites (Sampedro *et al*., 2001). In the yeast two‐hybrid system, AtXYL1, a natively secreted protein, is fused with a nuclear localized protein, GAL4 activation domain (AD). In general, the fusion of GAL4 AD transport targets protein to the nucleus, not through the endoplasmic reticulum and secretory pathway, so the N‐glycosylation that would occur in the endoplasmic reticulum might be disrupted. However, there are N‐linked glycoproteins present in the cytoplasm and nucleus (Hart and West, 2009), with evidence that certain glycoproteins return to the cytoplasm after entry into the ER or after secretion. (Chandra *et al*., 1998; Kung *et al*., 2009; Pedemonte *et al*., 1990; Reeves *et al*., 1981). Nonconventional soluble glycosyltransferases may exist in the cytoplasm or nucleus and directly modify the proteins in these compartments. Another possibility is that soluble N‐glycosylated proteins may be flipped across membranes or originated from secretory pathways (Varki *et al*., 2009). While we cannot rule out that the GRFT‐AtXYL1 interaction we discovered via the yeast two‐hybrid screen is the result of a direct protein‐protein interaction, direct evidence of glycan‐mediated interaction between the nicotiana homologue of AtXL1 and GRFT through the PLA assay lends support to the hypothesis that the interaction is indeed glycan‐mediated.

**Figure 5:**
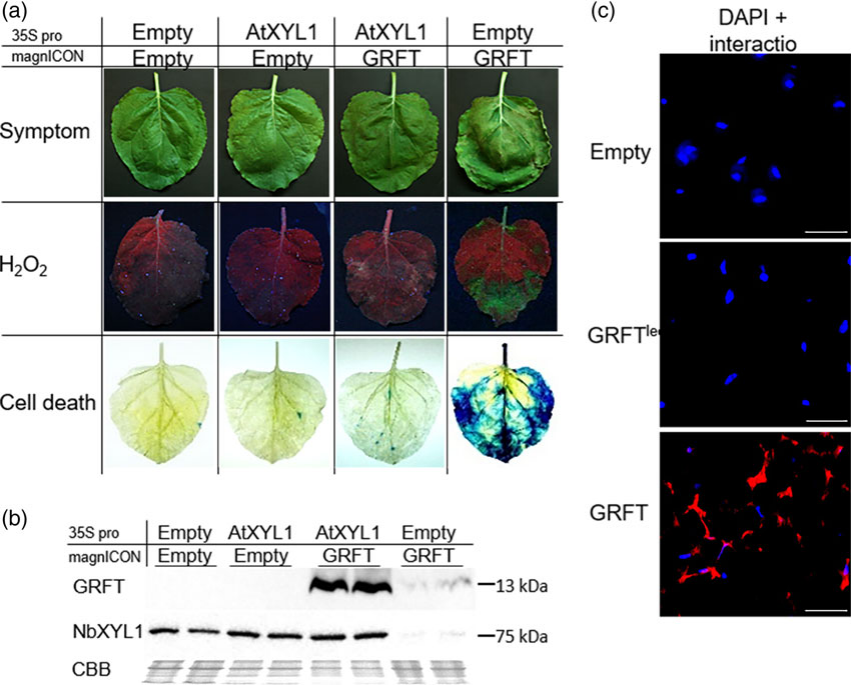
Interaction of XYL1 and GRFT‐induced HR‐like cell death in *N. benthamiana* plants. (a) Overexpression of AtXYL1 suppressed GRFT‐induced cell death in *N. benthamiana*. Detection of cell death and H_2_O_2_ generation in the inoculated plant leaves. Dead cells were stained by Evans blue, and H_2_O_2_ was detected using the H_2_O_2_‐sensitive fluorescent probe H2DCFDA. (b) Measurement of the GRFT accumulation levels by Western blotting. Anti‐GRFT (rabbit) and anti‐rabbit HRP were used for detection. CBB stain indicates loaded protein amounts are equal. (c) In situ association between GRFT and NbXYL1 in *N. benthamiana*. The plants were agro‐infiltrated with magnICON‐empty, magnICON‐GRFT and magnICON‐GRFT ^lec‐^, and the leaves were harvested 3 days after infiltration. The plant cell nuclei were visualized using DAPI staining (Blue). For detection of in situ molecular interaction, the Duolink in situ PLA kit was used. Two primary antibodies against GRFT (Goat) and NbXYL1 (rabbit) were used. With fluorescent microscopy, PLA‐red signals will be detected only when there is an *in vivo* interaction between GRFT and host protein. Scale bar: 50 μm. All test samples were measured in triplicate.

We investigated the effect of AtXYL1 on secreted GRFT‐mediated HR‐like cell death induction and GRFT accumulation level using 35S promoter‐driven AtXYL1 overexpression. The *N. benthamiana* plants where AtXYL1 was overexpressed showed delayed necrosis symptom development accompanied with H_2_O_2_ when secreted GRFT was co‐expressed (Figure 5a). In addition, overexpression of AtXYL1 strongly enhanced GRFT‐Apo accumulation, similar to what was observed in plants where NahG was co‐expressed with apoplast‐targeted GRFT (Figure 5b). The fact that AtXYL1 is a regulated factor for secreted GRFT‐induced necrosis is supported by XYL1 knock‐down experiments. XYL1 targeted pTRV‐based virus‐induced gene silencing (VIGS) accelerated the GRFT‐induced necrosis symptoms (Figure S9). We then identified the NbXYL1 sequence and created a specific antibody based on this NbXYL1 sequence. Interestingly, the plants in which only GRFT‐Apo was expressed showed reduction in NbXYL1 protein levels, and the reduction was rescued by AtXYL1 overexpression. AtXYL1 overexpression without co‐expression of GRFT did not affect NbXYL1 accumulated levels. This result strongly suggests that the physical interaction between GRFT‐Apo and NbXYL1 caused the XYL1 protein concentration reduction. We suggest that the presence of AtXYL1 sequesters GRFT‐Apo in a dominant‐negative fashion and thereby protects cell wall resident endogenous NbXYL1 from GRFT‐Apo‐mediated interaction, turnover and ultimately cell death.

We tried to verify the interaction between secreted GRFT and XYL1 *in vivo* by PLA assay. Only the plants expressing GRFT‐Apo, but not GRFT^lec‐^‐Apo or empty vector, displayed PLA signals (Figure 5c). This signal localized to the space outside the plant cells. These results indicate that GRFT‐Apo is interacting with XYL1 via its carbohydrate‐binding domain. It is reported that the N‐glycans in AtXYL1 have terminal mannose residues (Wildt and Gerngross, 2005). Mannose glycosylation pathways are conserved in plant and yeast, suggesting GRFT‐Apo can recognize XYL1 through the mannose N‐glycosylation in yeast cells and plant cells (Aebi, 2013). The interaction between GRFT‐Apo and XYL1 was confirmed by PLA assay using purified GRFT protein (Figure S10). PLA assay showed purified GRFT interacts with NbXYL1 in the apoplast. We speculate that this GRFT–host protein interaction plays an important role in its HR‐like cell death induction.

We also analysed XYL1 interaction with other mannose binding lectins. The overexpression of AtXYL1 prevented HR‐like cell death when secreted SP‐D or hMBL was expressed in *N. benthamiana*. The PLA assay showed that SP‐D and hMBL also interacted with NbXYL1. Surprisingly, SP‐D and hMBL accumulation levels were drastically suppressed by AtXYL1 overexpression. In contrast to SP‐D and hMBL, CV‐N‐induced HR‐like cell death was not altered by AtXYL1 overexpression, and an interaction between CV‐N and NbXYL1 could not be detected (Figure S12). We speculate that CV‐N interaction with another distinct host apoplast‐located protein may cause the SA‐mediated cell death phenomenon. Further understanding of this phenomenon could lead to a generalized strategy to enhance expression of lectins of pharmaceutical and industrial interest in plants.

## Discussion

In this study, we demonstrated that GRFT‐Apo induces HR‐like cell death accompanied by increased PR1 expression and generation of ROS in *N. benthamiana*. GRFT‐Apo‐induced cell death was dependent on salicylic acid (SA)‐mediated signalling. Our data prove that HR‐like cell death induction is regulated by the interaction between GRFT and host proteins, specifically the key host protein, XYL1. GRFT inhibits HIV entry and infection by binding to HIV envelope glycoprotein, gp120 (Mori *et al*., 2005). Abrogating the activity of GRFT in the lectin activity‐deficient mutant GRFT^lec‐^ not only eliminated its biochemical and pharmacological activities, but also eliminated GRFT's ability to interact with XYL1.

GRFT, SP‐D, CV‐N and hMBL are all mannose‐binding lectins with diverse pharmacological activities: SP‐D inhibits cell proliferation, migration and invasion by suppression of epidermal growth factor signalling (Hasegawa *et al*., 2015); CV‐N is known to block entry of the human immunodeficiency virus (HIV) into human cells (Boyd *et al*., 1997); hMBL binds and neutralizes influenza A virus and reduces the virus’ infection of respiratory epithelial cells (Hartshorn *et al*., 1993; Reading *et al*., 1997). hMBL works as a therapeutic microbicide against Ebola virus (Michelow *et al*., 2011). Like GRFT, their pharmaceutical activities depend on their mannose binding activities (Ferguson *et al*., 1999; Larsen *et al*., 2004; Liu *et al*., 2009). Several lectins that bind high mannose glycans, including GRFT and CV‐N, accumulate well in various plants such as tobacco, marshmallow, soya bean and rice, and do not cause necrosis symptoms (Drake *et al*., 2013; O'Keefe *et al*., 2009, 2015; Vamvaka *et al*., 2016). These studies support our data that demonstrate that cytosolic‐targeted expression of lectins do not induce necrotic responses in the host plant.

Here, we showed that similar to GRFT, SP‐D, CV‐N and hMBL induced SA‐dependent HR‐like cell death when they accumulated in apoplast. We showed the mutations which impair these lectins’ high‐mannose N‐glycan binding abilities prevented induction of cell death. These results suggested GRFT and other mannose binding lectins induced the same type of SA‐dependent cell death and their high‐mannose N‐glycan binding ability is responsible for the induction. However, GRFT, CV‐N and hMBL have shown little inflammatory effects or ROS generation induction in mammalian cells (Buffa *et al*., 2009; Nelson *et al*., 2014; O'Keefe *et al*., 2009). Only SP‐D induces oxidative burst and leads to apoptosis in human cells (Mahajan *et al*., 2013). These facts indicate the lectins might induce HR‐like cell death by interacting with a specific plant glycoprotein or activation of plant‐specific immune responses through their high‐mannose binding activities.

Using GRFT as bait in a yeast two‐hybrid screening system, AtXYL1 was identified as a key host factor with which GRFT interacts. In the context of the yeast two‐hybrid assay, we cannot tell whether the interaction between GRFT ‘bait’ and the AtXYL1 fragment ‘prey’ is mediated through the lectin activity of GRFT binding a AtXYL1 glycan expressed in the yeast system, or whether the interaction is at the amino acid level. There is precedent for N‐glycosylation of nuclear proteins (Chandra *et al*., 1998; Kung *et al*., 2009; Pedemonte *et al*., 1990; Reeves *et al*., 1981; Varki *et al*., 2009), and we speculate that the AtXYL1 fragment was directed to the secretory pathway through its own signal peptide in the yeast host and redirected to the nucleus, permitting interaction with GRFT through an N‐linked glycan. The PLA demonstrates that the interaction between GRFT and NbXYL1 requires lectin activity *in planta*.

**Figure 6:**
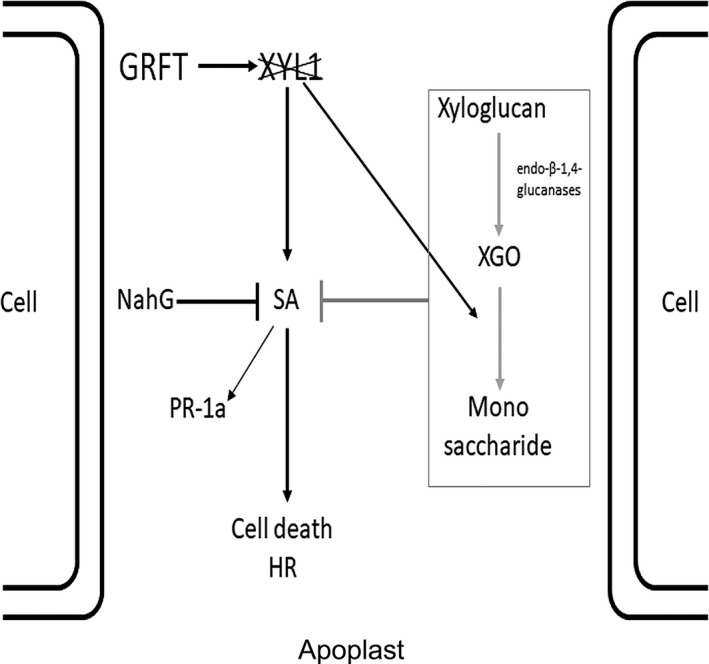
A schematic model of interaction of GRFT and XYL1 in tobacco plants. When secreted GRFT expressed in *N. benthamiana* plants, GRFT binds to XYL1 and reduces XYL1 accumulation level, which might lead to the inhibition of xyloglucan (XGO) degradation into monosaccharide. *N. benthamina* plants showed HR‐like cell death, accompanied with SA signalling. NahG, SA accumulation suppressor, rescue the *N. benthamiana* plants from HR and can accumulate GRFT in plants. The overexpression of the pepper xyloglucan‐specific endo‐β‐1,4‐glucanases inhibitor protein (CaXEGIP1) induced HR‐like cell death accompanied with salicylic acid‐dependent PR gene expressions cell death in *N. benthamiana* (Choi *et al*., 2013). Our study and Choi *et al*., 2013 strongly suggests Xyloglucan metabolism inhibition might be involved in SA‐dependent HR‐like cell death induction. Grey box and arrow bar are showing the Xyloglucan degradation pathway to monosaccharide (Shigeyama *et al*., 2016).

We showed that secreted GRFT interacted with NbXYL1 in the apoplast in *N. benthamiana*. Other mannose binding lectins, SP‐D and hMBL also interacted with NbXYL1, but CV‐N did not. Xyloglucan (XyG) is a ubiquitous plant polysaccharide, a component of hemicellulose in most dicot species, which cross‐links cellulose microfibrils in the wall and builds a cellulose‐XyG network in the cell wall (Somerville *et al*., 2004). XyG structure in cell walls is dynamic; it undergoes structural maturation and degradative turnover by apoplastic plant enzymes (Frankova and Fry, 2013). Plant endoglucanases including endo‐β‐1,4‐glucanases reduce the XyG polymer chain length and degrade XyG into oligosaccharides (XGO, Shigeyama *et al*., 2016). Apoplast localized enzymes including XYL1, a galactosidase (BGAL10), and a fucosidase (AXY8) are involved in XGO degradation into its various monosaccharides (Gunl and Pauly, 2011; Gunl *et al*., 2011; Sampedro *et al*., 2001, 2010, 2012). Recently, it was reported that *agrobacterium*‐mediated overexpression of the pepper xyloglucan‐specific endo‐β‐1,4‐glucanases inhibitor protein (CaXEGIP1) induced cell death in *N. benthamiana* (Choi *et al*., 2013). The HR‐like cell death was accompanied with salicylic acid‐dependent PR gene expression indicating XyG metabolism might be involved in the induction of the process (Choi *et al*., 2013). Our results showed XYL1 is a negative regulator of secreted GRFT‐induced HR‐like cell death (Figure 6). It is possible that AtXYL1 protects NbXYL1 by simply sequestering apoplast‐targeted GRFT. It is also possible that GRFT‐NbXYL1 complexes induce HR, while GRFT‐AtXYL1 complexes do not. Interestingly, secreted GRFT accumulation reduced levels NbXYL1, and we also saw this phenomenon in plants overexpressing secreted SP‐D and hMBL. We demonstrated that NbXYL1 interacts with SP‐D and hMBL in the apoplast of *N. benthamiana*, and overexpression of AtXYL1 reversed HR‐like cell death induced by secreted SP‐D and hMBL. Furthermore, a XYL1 knock‐down in *N. benthamiana* or Arabidopsis xyl1 knockout mutant under normal growing condition do not show cell death (Sampedro *et al*., 2010). We speculate that secreted lectins may impact other apoplast localized XGO degradation enzymes in addition to XYL1 and inhibit XyG metabolism, leading to HR‐like cell death as shown in CaXEGIP1 overexpression (Figure 6).

We do not yet fully understand why only GRFT, SP‐D and hMBL protein accumulation levels were enhanced by NahG expression while CV‐N accumulation levels seemed unaffected. Reports show that recombinant production of CV‐N in several systems including bacteria and transgenic plants resulted in relatively low protein yields (Colleluori *et al*., 2005; Gao *et al*., 2010; Sexton *et al*., 2006; Xiong *et al*., 2010). Recent research showed SA is involved in germination, photosynthesis, respiration, flowering and senescence (Rivas‐San Vicente and Plasencia, 2011). In fact, NahG expression enhances plant growth associated with enlarged cell size and extensive endoreduplication (Scott *et al*., 2004; Xia *et al*., 2009). Due to the combination of CV‐N instability in plant cell and cellular environmental changes brought on by NahG, CV‐N protein accumulation level might not be increased in plants where NahG is expressed, even when the plants were rescued from cell death.

In many plant species, lectins are reported to be involved in defence responses to pathogen infection (Peumans and Van Damme, 1995). Plant lectins interact with the glycoproteins on the surfaces of fungi, leading to pathogen growth inhibition (Broekaert *et al*., 1989; Fliegmann *et al*., 2004). Moreover, it has been reported that Arabidopsis lectins inhibit the systemic spread of plant viruses (Chisholm *et al*., 2000; Yamaji *et al*., 2012). With reports that lectins have potent antiviral activity and may also be useful in cancer diagnostics and therapeutics, there is likely to be interest in developing scalable manufacturing systems (Fu *et al*., 2011; Liu *et al*., 2009). We have shown some of the mechanisms that impact accumulation of GRFT and the other two lectins in plants. Clearly suppression of the SA pathway can rescue expression as this is commonly involved in the necrosis response to lectin expression. Similarly, it may be possible to overexpress the host ‘off‐target’ binder protein, such as XYL1, to relieve the necrosis and enhance accumulation.

In general, HR is a resistance response induced by interaction with plant resistance gene (R gene) and an avirulence factor of a pathogen (Avr gene) in plant. However, our data showed GRFT and other lectins including SP‐D, CV‐N and hMBL fused with apoplast signal expression directly induced HR‐like cell death in the absence of factors derived from pathogens. Our data are consistent with the research reported by a group who showed that pepper mannose‐binding lectin (CaMBL1) causes SA‐dependent cell death when CaMBL1 is overexpressed (Hwang and Hwang, 2011). It is possible that exogenous high mannose lectins located to the apoplast phenocopy a plant pathogen innate immune response induced by a pathogenic lectin.

Engineering plant hosts for biomanufacturing or ‘pharming’ of high value proteins is an emerging strategy, best demonstrated by efforts to ‘humanize’ plant glycosylation machinery (Daskalova *et al*., 2010; Grill *et al*., 2005). By characterizing the molecular basis of the necrosis response associated with secretion of mannose targeted lectins, we have demonstrated practically useful strategies to enhance expression of GRFT‐Fc lectibodies, BSA fusions or other engineered proteins by suppression of the SA expression pathway, or by augmenting levels of a target protein that is more rapidly metabolized in complex with the lectin.

## Experimental procedures

### Expression vector construct and plant growth conditions

The magnICON (Icon Genetics GmbH, Germany) system was used to express wild‐type griffithsin (GRFT), surfactant protein D (SP‐D), cyanovirin‐N (CV‐N), human mannose binding lectin (hMBL), galectin‐9 (Gal‐9), GRFT^lec‐^(D30A, D70A and D112A), SP‐D^lec‐^(E321Q and N323D), CV‐N^lec‐^(K3N, Y7A, E23I and N93A) and hMBL^lec‐^(G51E, R52C, G54D, G57E and G60E), respectively (Chang and Bewley, 2002; Larsen *et al*., 2004; Ogasawara and Voelker, 1995; Xue *et al*., 2012). The optimized lectin sequences for *Nicotiana benthamiana* were synthesized at Integrated DNA Technologies (IDT). The synthetic encoding GRFT and GRFT^lec‐^ cDNA were cloned into magnICON vector pICH11599 in the NcoI and SacI restriction enzyme sites. NcoI and PstI sites were used to clone SP‐D, CV‐N, hMBL and Gal‐9 and lectin‐deficient mutant fragments (each lectin containing C‐terminal 6× His tag) into pICH11599.

The NahG sequence, which was optimized for *N. benthamiana* plants, was synthesized at Integrated DNA Technologies (IDT) and cloned into pICH38077 PVX‐based magnICON vector using the BamHI and SacI sites. *Nicotiana benthamiana* were used in these experiments. Seeds were directly sown in peat pellets and grown at 26 °C with a 16‐h photoperiod.

### Viral vector‐based expression of GRFT in *N. benthamiana*

*Agrobacterium* vacuum infiltration was performed as described in Matoba *et al*. (Matoba *et al*., 2010). pICH11599‐GRFT, pICH14011 integrase promodule, pICH17388 apoplast module (Marillonnet *et al*., 2004), pICH38077‐NahG (PVX one component expression vector) and pRI101‐AN vector (for AtXYL1 overexpression, Clontech) were transformed into *Agrobacterium tumefaciens* GV3101 by electroporation. Each *Agrobacterium* culture was suspended in infiltration buffer (10 mm MES, 10 mm MgSO4, pH 5.5) and mixed at OD_600_ 0.1. The *Agrobacterium* suspension was infiltrated into leaves using vacuum pump. At 5 days postinfiltration (dpi), infected leaves were harvested and analysed.

### Detection of cell death and H_2_O_2_

The leaves of *N. benthamiana* were submerged in 0.25% Evans blue (Alfa Aesar) solution for 10 min under vacuum. The leaves were then washed three times with water and destained in 70% ethanol for 2 weeks. H_2_O_2_ generation in leaf tissues was detected using 2′7′‐dichlorofluorescein diacetate (H2DCFDA, Sigma‐Aldrich, Kim *et al*., 2008). Briefly, leaves were reacted with loading buffer (10 mm Tris‐HCl and 50 mm KCl, pH 7.2) containing 1 mm H2DCFDA under vacuum infiltration for 5 min. The samples were incubated in the dark for 20 min at room temperature, and washed two times in loading buffer. Fluorescence was observed under the UV lamp (365 nm).

### Quantitative real‐time RT‐PCR

Total RNA was isolated using Trizol reagent (Invitrogen) according to the manufacture's protocol. For the real‐time RT‐PCR, 1 μg of total RNA was used as a template for cDNA synthesis. The first‐strand cDNA was synthesized by the Takara RNA PCR Kit with a random primer. Real‐time RT‐PCR analysis was performed using the iQ5 Real‐Time PCR Detection System (Bio‐Rad, Hercules, CA, USA). Real‐time RT‐PCR was carried out using 1 μL of the RT reaction mixture and SYBR Green mixture (Bio‐Rad). For the amplification of the *N. benthamiana* PR1 genes, the following primer pairs were used (Table S1): NbPR1F (5′‐CCCTCCCACATGTCATTCTT ‐3′) and NbPR1R (5′‐ATTTCTCGCTCAGCTGTGGT ‐3′). For real‐time PCR, the actin gene was used as an internal control using primer pair NbActinF (5′‐ATGCGCAAAATTATGCTTCC ‐3′) and NbActinR (5′‐TCTCATCGACCCACATCTCA ‐3′). PR1 gene expression was normalized to actin.

### GRFT detection by enzyme‐linked immunosorbent assay

ELISA was performed according to the method of Boyd *et al*. (Boyd *et al*., 1997). Briefly, a microtitre plate (NUNC, Nalgene, Thermo Scientific, Waltham, Massachusetts, USA) was coated with HIV gp120 (Protein Sciences Corp., Meriden, CT). Plates were blocked with 5% PBSTM (phosphate‐buffered saline [pH 7.4], 0.05% [vol/vol] Tween‐20, 5% nonfat dry milk) for 1 h at room temperature. Total proteins extracted from the plants agro‐infiltrated with magnICON were incubated in each well. As a standard, recombinant GRFT purified from *N. benthamiana* was diluted twofold from 200 to 0.78 ng GRFT/well. Positive controls and *N. benthamiana* plants extracted protein were incubated as 50 μL volumes. Rabbit polyclonal anti‐GRFT diluted 1:1000 in PBS was added to the wells followed by goat anti‐rabbit HRP‐conjugated antibody (Sigma‐Aldrich) 1:2500 in PBS. Wells were washed (twice after coating and blocking and four times after sample and antibody addition) with PBS supplemented with 0.1% Tween‐20 (Merck, Darmstadt, Germany), and incubation periods were 2 h at 37 °C or overnight at 4 °C. TMB substrate (tetramethylbenzidine, Sigma‐Aldrich) was added in 50 μL to each well. The reaction was stopped with equal volumes of 1 m H_2_SO_4_, and the absorbance was measured at OD_450_.

### Western blot analysis

Total proteins from *N. benthamiana* plants, inoculated with wild‐type and mutant lectins, were extracted with 30 mm acetate buffer (pH 4.0, containing 313 mm NaCl, 23 mM ascorbic acid, and 8 mm sodium metabisulphite) as described by O'Keefe *et al*. (O'Keefe *et al*., 2009). The samples were boiled for 10 min, separated on a 12% SDS–PAGE gel (Bio‐Rad) and transferred to a membrane (Trans‐Blot Turbo PVDF Transfer Pack, Bio‐Rad, Richmond, CA). The membranes were blocked with 5% PBSTM (phosphate‐buffered saline [pH 7.4], 0.05% [vol/vol] Tween‐20, 5% nonfat dry milk) for 1 h at room temperature, incubated with anti‐GRFT rabbit antibody overnight at 4 °C, and then rabbit IgG for 1 h at room temperature. For detection of the signal, we used the ECL Western blot system. For detection of SP‐D, CV‐N, hMBL and Gal‐9, the anti‐his antibody (Abcam) and the anti‐rabbit HRP were used as primary and secondary antibody respectively.

### Protein infiltration

GRFT and GRFT^lec‐^ were purified as previously described (O'Keefe *et al*., 2009) and were >99% pure. *N. benthamiana* leaves were infiltrated with 0.9 mg/mL GRFT or GRFT^lec‐^ using a vacuum pump. Five days after the infiltration, Evans blue staining and H_2_O_2_ detecting were performed.

### In situ PLA assay

magnICON‐GRFT and GRFT^lec‐^ agro‐infiltrated *N. benthamiana*’ leaves were harvested 5 days after the infiltration. The leaves were fixed with 4% paraformaldehyde, and tissue embedding was performed according to the protocol (Javelle *et al*., 2011). The sectioning and fixing onto slide glasses were carried out by Research cores service (University of Louisville). The fixed leaf samples were incubated with GRFT rabbit antibody and *N. benthamiana* total protein mouse antibody or secreted protein mouse antibody, with GRFT goat antibody and NbXYL1 rabbit antibody. PLA was performed according to the manufacturer's protocol using the Duolink Detection Kit (Sigma‐Aldrich). Proximity signal was detected with Axio Observer Z1 microscope (Carl Zeiss, Thornwood, NY).

Total protein extracts were prepared from *N. benthamiana* by grinding the tissue using extraction buffer (20 mm NaAc, 50 mm NaCl, 1% Triton‐X 100, pH7.0). Secreted protein extracts were prepared by vacuum infiltrating *N. benthamiana* leaves with Tris‐NaCl buffer (20 mm Tris, 100 mm NaCl, 20 mm sodium acetated, 4 mm sodium metabisulphite, pH7.0). Buffer inoculated leaves were spun at 2000 ***g*** for 15 min. Centrifuged protein was collected into 1.5‐mL tubes. Antibodies against total and secreted proteins from *N. benthamiana* were produced in mice by injection of 50μg/50μL of each of the protein preparations five times. After all subsequent injections, mouse sera were used for detection.

For identifying the N‐terminus sequence of NbXYL1, we used *N. sylvestris* XYL1 (XM009800839) sequence which is known. This sequence has 100% homology Fdewith NbXYL1. The cDNA generated using the primers (NbXYLF:5′‐GGTTGGTACCATGGATTTGTATGGTTCACATCCAATGTATA‐3′, NbXYL1R:5′‐ AGCCGAATTCCTCAAGGTGAAATCCCCATTTTCC‐3′) was cloned using TA cloning system (Promega). After sequencing seven clones, the sequence of NbXYL1 was determined.

### Statistical analyses

Data are presented as the mean ± SEM; the number of observations (*n*) refers to technical replicates. Data analysis was carried out by either an unpaired Student's *t*‐test or one‐way analysis of variance with Bonferroni's multiple comparison test (MCT), as indicated in the text.

## Supporting information


**Figure S1** GRFT accumulation in the apoplast induced an HR‐like cell death response.
**Figure S2** Schematic representation of MagnICON promodule vectors used for GRFT and NahG expression.
**Figure S3** GRFT accumulation in the apoplast induced an HR‐like cell death response using binary vector.
**Figure S4** NahG and GRFT^lec‐^ did not induce cell death in *N. benthamiana*.
**Figure S5** Vacuum infiltration of purified GRFT protein induces HR‐like cell death in *N. benthamiana*.
**Figure S6** Expression of galactoside‐binding protein, Galectin‐9 (Gal‐9) and mannose binding lectins with apoplast signal in *N. benthamiana* plants.
**Figure S7** Expression level of lectin deficients of GRFT, SP‐D, CV‐N, hMBL and Gal‐9 inhibits the cell death in *N. benthamiana*.
**Figure S8** In situ association between GRFT and membrane protein in *N. benthamiana*.
**Figure S9** GRFT induced severe necrotic symptom in NbXYL1 silenced *N. benthamiana* plants using pTRV‐based VIGS.
**Figure S10** In situ association between GRFT and NbXYL1 in *N. benthamiana*.
**Figure S11** Immunostaining using single antibody.
**Figure S12** Interaction of XYL1 and lectins in *N. benthamiana* plants.
**Table S1** Primers and applicant characteristics for RT‐qPCR.
