# Supplementary material for: Characterization of the hypersensitive response‐like cell death phenomenon induced by targeting antiviral lectin griffithsin to the secretory pathway
Source: Plant Biotechnol J. 2018 May 2;16(10):1811–21. doi: 10.1111/pbi.12917 (PMC6131415; doi:10.1111/pbi.12917)
Supplement: Supplementary file 1 — Figure S1 GRFT accumulation in the apoplast induced an HR‐like cell death response. Figure S2 Schematic representation of MagnICON promodule vectors used for GRFT and NahG expression. Figure S3 GRFT accumulation in the apoplast induced an HR‐like cell death response using binary vector. Figure S4 NahG and GRFTlec‐ did not induce cell death in N. benthamiana. Figure S5 Vacuum infiltration of purified GRFT protein induces HR‐like cell death in N. benthamiana. Figure S6 Expression of galactoside‐binding protein, Galectin‐9 (Gal‐9) and mannose binding lectins with apoplast signal in N. benthamiana plants. Figure S7 Expression level of lectin deficients of GRFT, SP‐D, CV‐N, hMBL and Gal‐9 inhibits the cell death in N. benthamiana. Figure S8 In situ association between GRFT and membrane protein in N. benthamiana. Figure S9 GRFT induced severe necrotic symptom in NbXYL1 silenced N. benthamiana plants using pTRV‐based VIGS. Figure S10 In situ association between GRFT and NbXYL1 in N. benthamiana. Figure S11 Immunostaining using single antibody. Figure S12 Interaction of XYL1 and lectins in N. benthamiana plants. Table S1 Primers and applicant characteristics for RT‐qPCR. [file PBI-16-1811-s001.docx]

**Supporting Information**


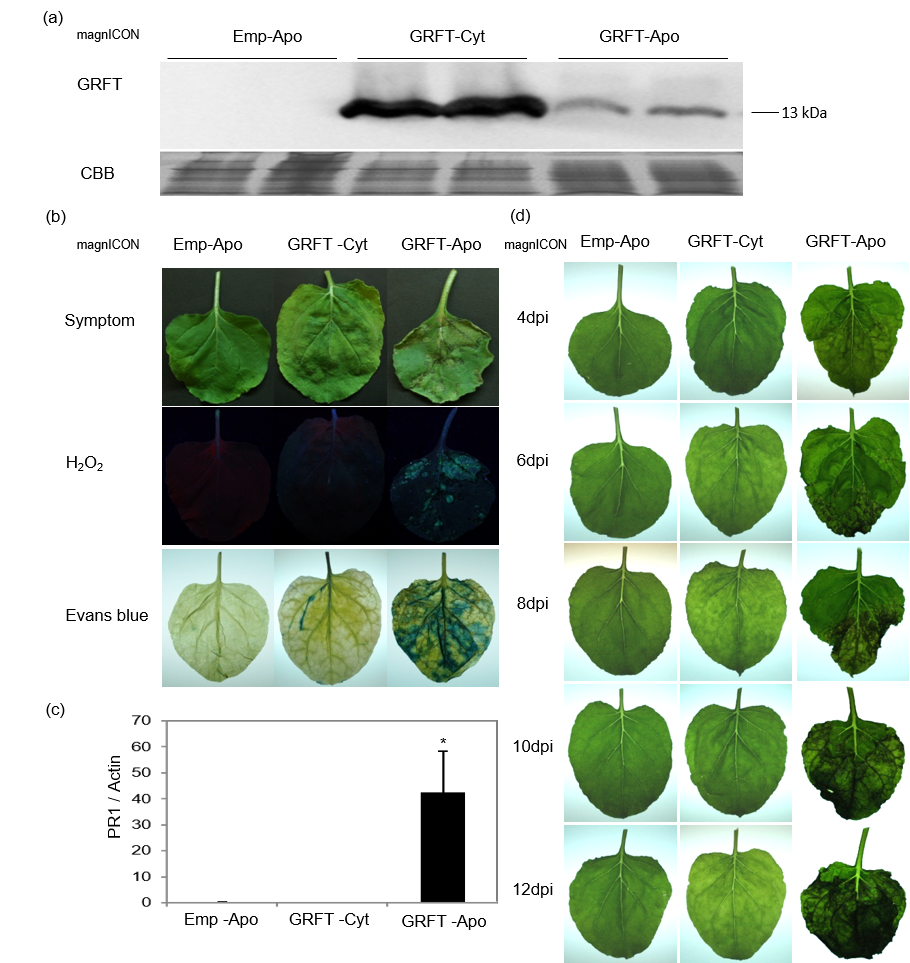


Figure S1

GRFT accumulation in the apoplast induced an HR-like cell death response.

(a) Western blot of total soluble protein extracted from plants expressing pICHgrft-Cyt and pICHgrft-Apo 5 days after the inoculation (dpi). CBB stain indicates loaded protein amounts are equal. (b) *N. benthamiana* plants were infiltrated with pICHgrft-Cyt or pICHgrft-Apo. H_2_O_2_ generation was analysed by using the H_2_O_2_-sensitive fluorescent probe H2DCFDA while cell death was confirmed by Evans blue staining. (c)**.** Analysis of PR1 gene expression levels was performed by realtime RT-PCR (B-C). The PR1 mRNA levels relative to the actin mRNA levels are presented. *P<0.05, asterisks indicate significant difference [Oneway ANOVA with Bonferroni’s multiple comparison test (n=3)] between inoculated plants group. Error bars represent standard errors of the means. The analysis was done 5 days after the inoculation. (d) The secreted GRFT accumulation induced a necrotic symptom in *N. benthamiana*. Three biological repeats were performed showing the same result in all instances. Results for one of these biological repeats are presented for days 4, 6, 8, 10 and 12 post infiltrations.


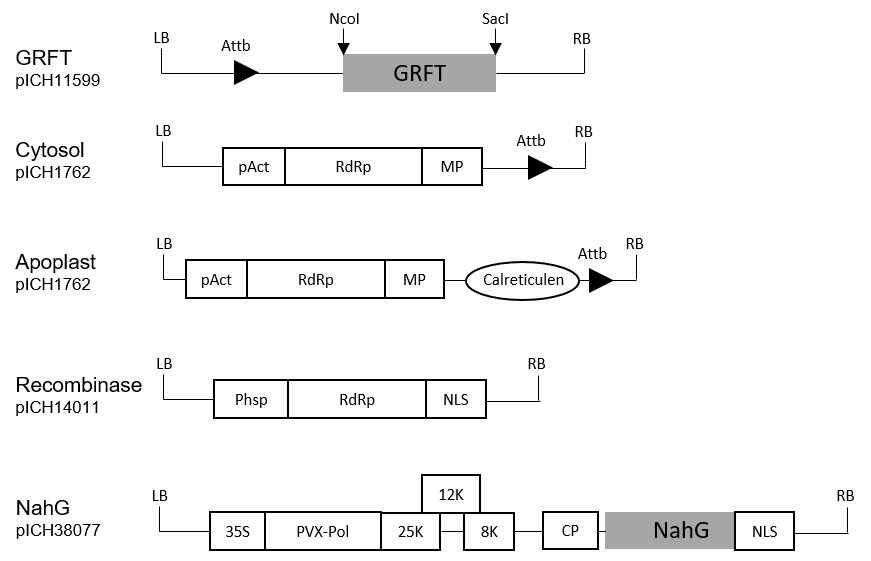


Figure S2

Schematic representation of MagnICON promodule vectors used for GRFT and NahG expression. The synthesized native GRFT was inserted into pICH11599. The pICH11599-GRFT (magnICON-GRFT) was combined with either a cytosol targeting promodule (pICH17388) or with the promodule (pICH1762) that contains the calreticulen apoplast targeting signal. Recombinase between promodules was facilitated by a *Streptomyces* phage C31 integrase encoded by the PhiC31 gene carried on pICH14011. pICH38077 is a PVX one component expression vector. The attB site is the recombinase recognition site. pAct is a fragment from the Arabidopsis actin 2 (ACT2) promoter. MP is the movement protein of the tobacco mosaic virus (TMV). RdRp is the RNA-dependant RNA promoter. Phsp is promoter of Arabidopsis gene encoding for heat shock protein hsp 81.1. PVX-Pol is polymerase/replicase from Potato virus X (PVX). CP is PVX coat protein. NLS is nuclear localization signal.


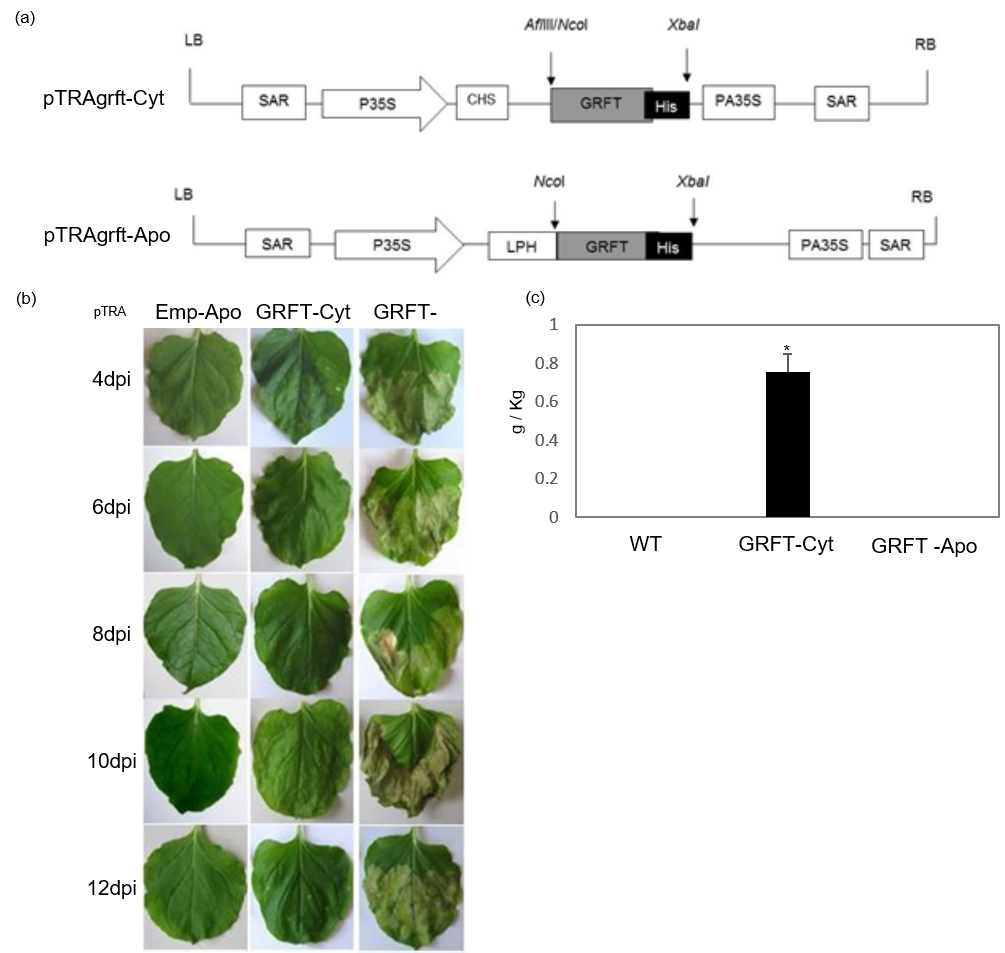


Figure S3

GRFT accumulation in the apoplast induced an HR-like cell death response using binary vector. (a) Schematic representation of pTRA vectors used for GRFT expression. GRFT was cloned into the *Afl*II-*Xba*I site of pTRAc (cytosol targeting) resulting in pTRAgrft-Cyt. To generate pTRAgrft-Apo an *Nco*I-*Xba*I GRFT PCR product was cloned. LPH is the plant codon optimized leader peptide derived from murine heavy chain of mAb24. His represents the hexa histidine-tag. (b) The secreted GRFT accumulation induced a necrotic symptom in *N. benthamiana*. Three biological repeats were performed showing the same result in all instances. Results for one of these biological repeats are presented for days 4, 6, 8, 10 and 12 post infiltrations. (c) Measurement of GRFT accumulation levels by ELISA. HIV gp-120 was bound to the wells of a 96-well plate and subsequently incubated with total protein from the plants agro-infiltrated with pTRAgrft-Cyt or pTRAgrft-Apo. After visualized by HRP-labeled anti-GRFT antibody, OD was measured by absorbance at 450 nm. *P<0.05, asterisks indicate significant difference [Oneway ANOVA with Bonferroni’s multiple comparison test (n=3)] between inoculated plant and WT plants. Error bars represent standard errors of the means.


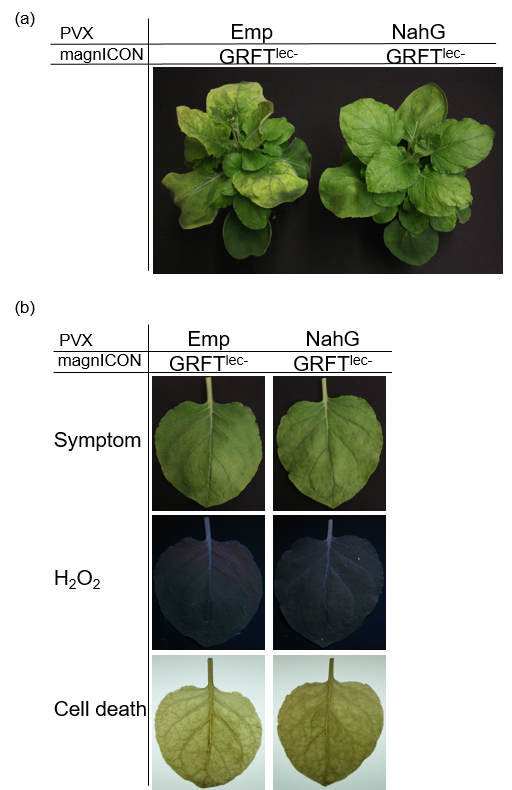


Figure S4

NahG and GRFT^lec-^ did not induce cell death in *N. benthamiana*. (a) Co-expression with PVX-empty + magnICON-GRFT^lec-^ and PVX-NahG + magnICON-GRFT^lec-^ were vacuum agro-infiltrated with *N. benthamiana* and the images were captured 5 days after the infiltration. (b) Detection of cell death and H_2_O_2_ generation in the inoculated plant leaves. Dead cells were stained by Evans blue and H_2_O_2_ was detected using the H_2_O_2_-sensitive fluorescent probe H2DCFDA.


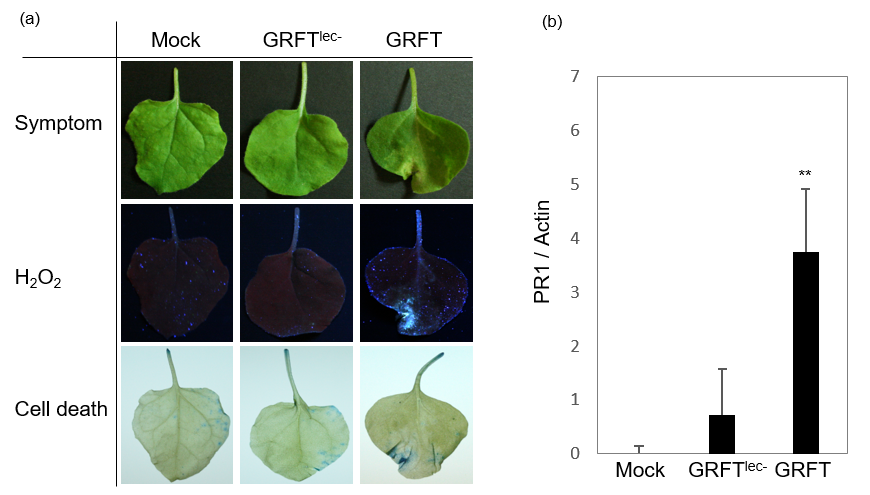


Figure S5

Vacuum infiltration of purified GRFT protein induces HR-like cell death in *N. benthamiana*. (a) PBS buffer, purified GRFT^lec-^ protein or purified GRFT protein were vacuum infiltrated with *N. benthamiana*. Leaves were harvested 5 days after the infiltration and analyzed. Necrosis induction, H_2_O_2_ generation and cell death by GRFT were induced by the presence of purified GRFT protein expression in the *N. benthamiana*. Detection of cell death and H_2_O_2_ generation in the inoculated plant leaves. Dead cells were stained by Evans blue and H_2_O_2_ was detected using the H_2_O_2_-sensitive fluorescent probe H2DCFDA. (b) Analysis of PR1 expression levels by real-time RT-PCR. The PR1 mRNA levels relative to the actin mRNA levels were shown. ***P* < 0.01, asterisks indicate significant difference [Oneway ANOVA with Bonferroni’s multiple comparison test (n=3)] between inoculated plants group. Error bars represent standard errors of the means.


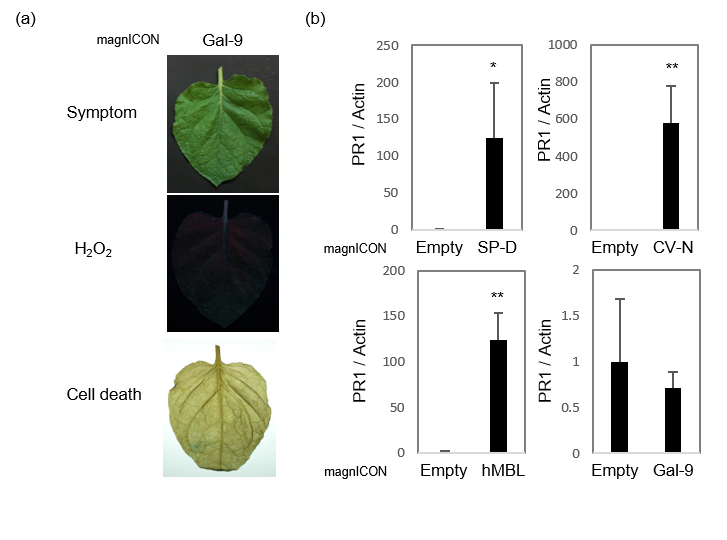


Figure S6

Expression of galactoside-binding protein, Galectin-9 (Gal-9) and mannose binding lectins with apoplast signal in *N. benthamiana* plants. (a) Detection of cell death and H_2_O_2_ generation in the inoculated plant leaves. Dead cells were stained by Evans blue and H_2_O_2_ was detected using the H_2_O_2_-sensitive fluorescent probe H2DCFDA. (b) Analysis of PR1 expression level by real-time RT-PCR. The PR1 mRNA levels relative to the actin mRNA levels were shown. SP-D, CV-N, hMBL or Gal-9 were vacuum agro-infiltrated with *N. benthamiana*. Leaves were harvested 5 days after the infiltration and analyzed. ***P* < 0.01 and *P<0.05, asterisks indicate significant difference [Student's t-test (n=3)] between inoculated plant groups. Error bars represent standard errors of the means. All test samples were measured in triplicate.


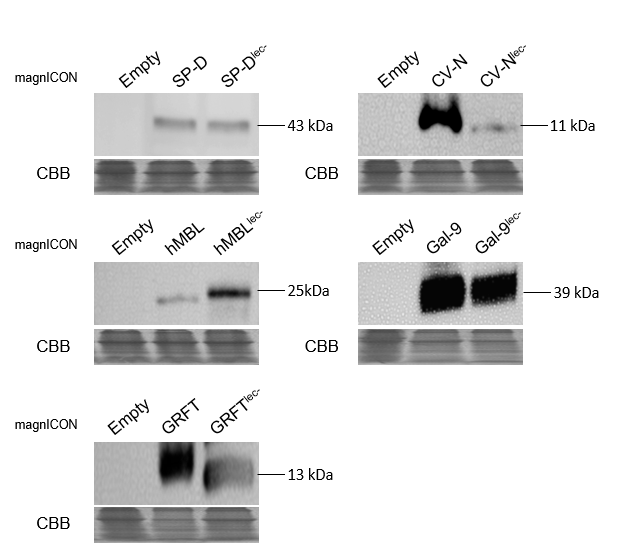


Figure S7

Expression level of lectin deficients of SP-D, CV-N, hMBL, Gal-9 and GRFT inhibits the cell death in *N. benthamiana*. Anti-His (rabbit) and anti-rabbit HRP were used for the western blotting. Empty magnICON vector, wild type and lectin deficient of SP-D, CV-N, hMBL, Gal-9 and GRFT were vacuum agro-infiltrated into *N. benthamiana*. Leaves were harvested 5 days after the infiltration and were extracted for western blotting. CBB stain indicates loaded protein amounts are equal.


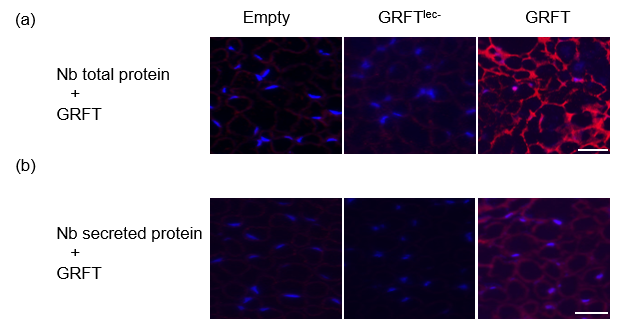


Figure S8

In situ association between GRFT and membrane protein in *N. benthamiana*. The plants were agro-infiltrated with magnICON-empty, magnICON-GRFT and magnICON- GRFT^lec-^, and the leaves were harvested 3 days after infiltration. The plant cell nuclei were visualized using DAPI staining (Blue). For detection of in situ molecular interaction, the Duolink in situ PLA kit was used. (a) Nb total protein and GRFT interation. Scable bar: 50 µm. (b) Nb secreted protein and GRFT interation. Two primary antibodies against GRFT and total protein of *N. benthamiana* or secreted protein of *N. benthamiana* were used. With fluorescent microscopy, PLA-red signals will be detected only when there is an in vivo interaction between GRFT and host protein. The signals for the interaction of GRFT-Nb tptal protein and GRFT-Nb secreted protein were detected in the apoplast. Scable bar: 50 µm. All test samples were measured in triplicate.


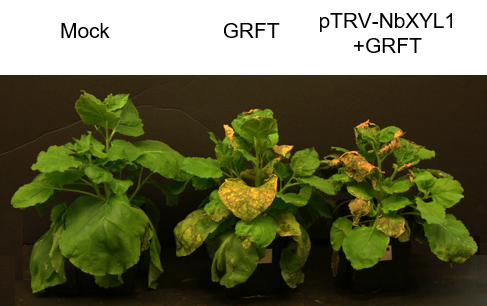


Figure S9

GRFT induced severe necrotic symptom in NbXYL1 silenced *N. benthamiana* plants using pTRV-based VIGS. 250bp of NbXYL1 sequence was inserted in pTRV vector (TAIR) using primer XYL1F (ACGGGGAATTCTGGCTGTATCTGCTCGTAATG) and XYL1R (GGAAACTCGAGCGCGCT GCATTGGTATTATG).


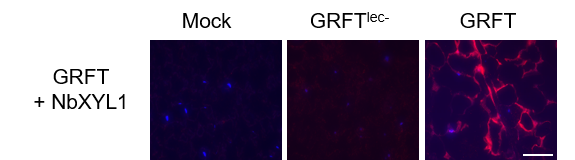


Figure S10

In situ association between purified GRFT and NbXYL1 in *N. benthamiana*. PBS buffer, purified GRFT^lec-^ protein or purified GRFT protein were vacuum infiltrated with *N. benthamiana*. Leaves were harvested 3 days after the infiltration and analyzed. The plant cell nuclei were visualized using DAPI staining (Blue). For detection of in situ molecular interaction, the Duolink in situ PLA kit was used. Two primary antibodies against GRFT (Goat) and NbXYL1 (rabbit) were used. With fluorescent microscopy, PLA-red signals will be detected only when there is an in vivo interaction between GRFT and host protein. Scable bar: 50 µm.


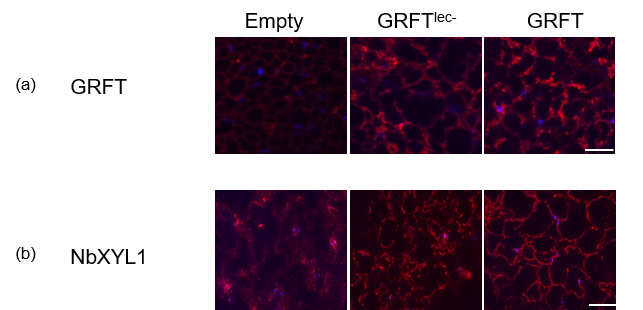


Figure S11

Immunostaining using single antibody. To demonstrate the qualities of Goat-GRFT antibody (a) and Rabbit-NbXYL1 antibody (b) used in the duolink system, we performed immunostaining. Scable bar: 50 µm.


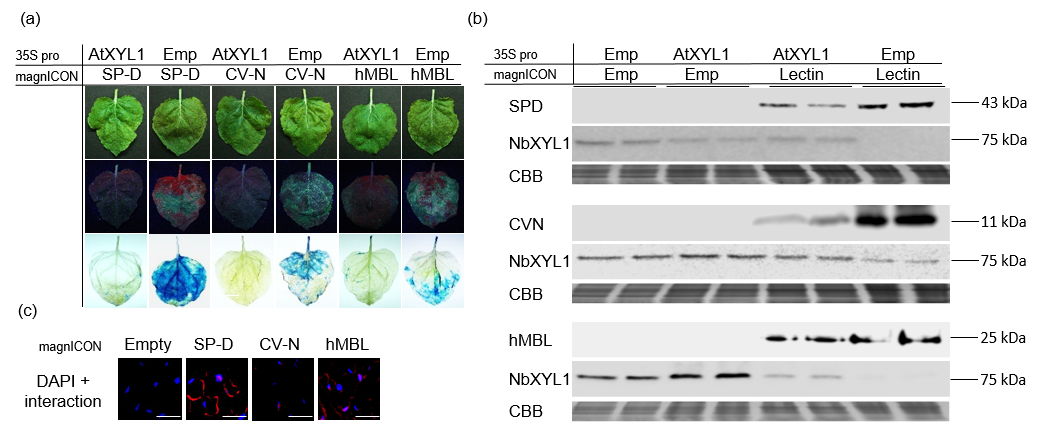


Figure S12

Interaction of XYL1 and lectins in *N. benthamiana* plants. (a) Overexpression of AtXYL1 suppressed mannose binding lectin-induced cell death in *N. benthamiana*. Detection of cell death and H_2_O_2_ generation in the inoculated plant leaves. Dead cells were stained by Evans blue and H_2_O_2_ was detected using the H_2_O_2_-sensitive fluorescent probe H2DCFDA. (b) Measurement of the lectins accumulation levels by western blotting. Anti-Flag (mouse) and anti-mouse HRP were used for detection. CBB stain indicates loaded protein amounts are equal. (c) In situ association between lectins and NbXYL1 in *N. benthamiana*. The plants were agro-infiltrated with magnICON-empty, magnICON-lectins, and the leaves were harvested 3 days after infiltration. The plant cell nuclei were visualized using DAPI staining (Blue). For detection of in situ molecular interaction, the Duolink in situ PLA kit was used. Two primary antibodies against Flag (mouse) and NbXYL1 (rabbit) were used. With fluorescent microscopy, PLA-red signals will be detected only when there is an in vivo interaction between GRFT and host protein. Scable bar: 50 µm. All test samples were measured in triplicate.

**Table S1 Primers and applicant characteristics for RT-qPCR.**

| **Gene** | **Gene Name** | **Accession Number** | **RT-qPCR Primer Sequences**  **Forward(F) and Reverse(R)** | **L** | **A** | **E(%)** | **R^2^** | **Slope** | **y-int** |
| --- | --- | --- | --- | --- | --- | --- | --- | --- | --- |
| PR-1a | Pathogenesis-related protein 1a | X06930 | F 5’-ATG CGC AAA ATT ATG CTT CC-3’  R 5’-TCT CAT CGA CCC ACA TCT CA-3’ | 160 | 138 | 105.2 | 0.970 | -3.2018 | 34.59 |
| Actin | *Nicotiana benthamiana* Actin | AY594294 | 5’-AGA TCC TCA CAG AGC GTG GT-3’  5’-TGG TAA TCA CTT GCC CAT CA-3’ | 343 | 542 | 100.4 | 0.999 | -3.311 | 23.97 |

List of genes used in RT-qPCR. The designed primer sequences, location of amplicon (L) and amplicon length (A). Validation of qPCR shown by efficiency (E), R^2^, slope and y-intercept (y-int) of the calibration curve. The calibration curve was obtained from the iQ5 Optical System Software Version 2.2. Primers were designed using PrimerQuest by Integrated DNA Technologies. (<https://www.idtdna.com/Primerquest/Home/Index>).
